# Prognositic value of CD73-adenosinergic pathway in solid tumor: A meta-analysis and systematic review

**DOI:** 10.18632/oncotarget.16905

**Published:** 2017-04-06

**Authors:** Rong Wang, Yingying Zhang, Xia Lin, Yalin Gao, Ying Zhu

**Affiliations:** ^1^ Department of Gynecology, Cancer Institute, Key Laboratory of Cancer Prevention and Intervention, National Ministry of Education, Provincial Key Laboratory of Molecular Biology in Medical Sciences, The Second Affiliated Hospital, Zhejiang University School of Medicine, Hangzhou 310009, China; ^2^ Department of Breast Surgery, First Affiliated Hospital of Zhengzhou University, Zhengzhou 450052, China

**Keywords:** CD73-adenosinergic pathway, overall survival, disease-free survival, solid tumor

## Abstract

CD73 is a glycosylphosphatidylinositol (GPI) anchored cell surface protein that is encoded by NT5E gene, plays multiple roles in tumor processes. Previous studies have presented a potential value of CD73 served as a detectable biomarker for prognosis of several solid tumors, but the results were more controversially. A comprehensive meta-analysis was conducted to precisely evaluate the prognostic role of CD73 in solid tumors. The included studies were searched in PubMed, Web of Science and EBSCO from Jan 1990 to Jan 2016. Pooled hazard ratios (HR) and corresponding 95% confidence intervals (CI) for overall survival (OS), disease free survival (DFS) were carried out using a fixed or random effects model. Totally, 13 studies about 12,533 patients were included. CD73-high expression was correlating with poor OS (pooled HR = 1.28, 95% CI = 1.19–1.37). In addition, CD73 expression had borderline association with worse DFS (pooled HR = 1.28, 95% CI = 1.01–1.62). Egger’s tests indicated that there was no evidence of significant publication bias. CD73 is an efficient prognostic biomarker in solid tumors, and over-expression of CD73 is associated with inverse OS or DFS. But this predictive value and target therapy for clinical practice yet needs advanced research.

## INTRODUCTION

CD73, also known as ecto-5′-nucleotidase (NT5E), is a glycosylphosphatidylinositol linked cell surface enzyme found in normal tissues. Originally, CD73 was defined as a lymphocyte differentiation antigen and an adhesion molecule for lymphocytes binding to endothelium [1]. Recent studies implied that CD73 was over-expressed on various kinds of solid malignant tumors (i.e., breast cancer [2], colorectal cancer [3], prostate cancer [4], ovarian cancer [5], and gallbladder cancer [6]). Over-expression of CD73 was driven by tumor hypoxic microenvironment and some soluble inflammatory factors, such as type I IFNs, TNF-α, IL-1β, TGF-β and Wnt activators [7]. Otherwise, in breast cancer, CD73 expression can also be found negatively regulated by estrogen receptor (ER) expression [8]. CD73 catalyzes the conversion of adenosine (ADO) from AMP released to the extracellular environment, and control a variety of physiologic responses, as well as the development of cancer [9].

**Figure 1:**
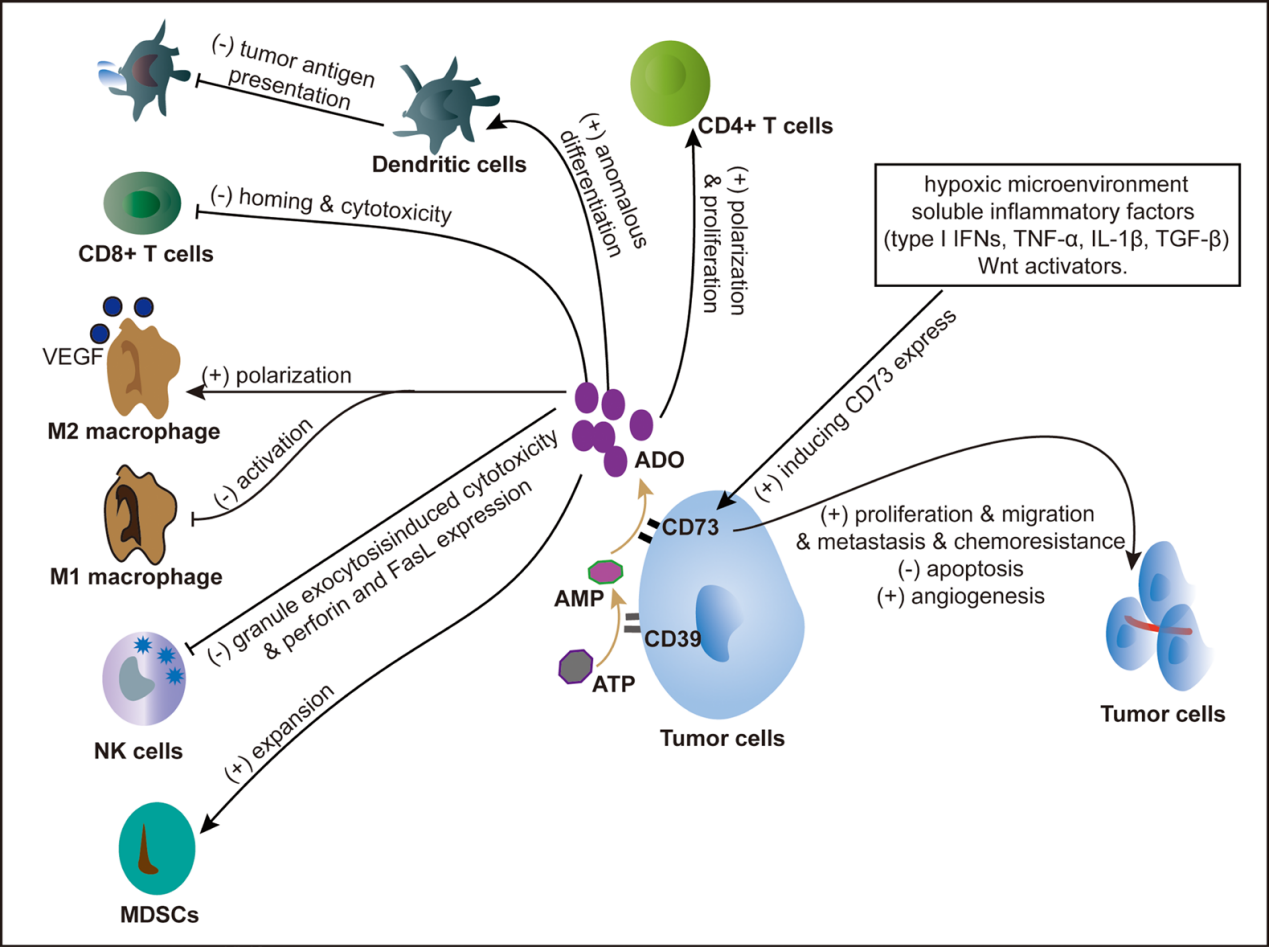
Overview of the CD73-adenosinergic pathway (**A**) Adenosine limiting cytotoxic activity of effective immune cells; (**B**) Adenosine enhancing immunosuppressive effects; (**C**) Adenosine inducing anomalous differentiation and weakening the function of antigen presenting cells; (D) CD73 promoting tumor cells proliferation, migration, angiogenesis, and chemoresistance. ADO: Adenosine, AMP: Adenosine Monophosphate, ATP: Adenosine Triphosphate, NK cells: Natural Killer Cells, MDSCs: Myeloid Derived Suppressor Cells, VEGF: Vascular Endothelial Growth Factor.

CD73 has both nonenzymatic and enzymatic functions in tumor environment [10]. For the nonenzymatic functions, CD73 plays a pivotal role for tumor cells proliferation, angiogenesis and apoptosis, via modulating extrinsic signaling, like EGFR/Akt, VEGF/Akt pathway and impairing antitumor immunity [11, 12]. CD73 endows tumor cells “invasive phenotype”, by reducing cell-cell adhesion, and inducing epithelial-mesenchymal transition (EMT) through the regulation of cadherin-1 and vimentin. So CD73 significantly contributes to tumor metastasis [7]. The enzymatic functions refer to CD73-generated adenosine playing an important role in tumor immune tolerance. Extracellular ADO can exert effect on tumor immune microenvironment through multiple pathways [1, 13]: 1) Limiting cytotoxic activity of effective immune cells: ADO significantly reduces CD8+ T cells homing and inhibits NK cells by interfering with the process of granule exocytosis and reducing the ability of NK cells to adhere to tumor cells [14, 15]. 2) Enhancing immunosuppressive effects: ADO inhibits M1 macrophage activation and induces M2 macrophage polarization via A2A and A2B receptors [16–18]. Myeloid-derived suppressor cells (MDSC) perform a vital role in damaging tumor immune surveillance. ADO promotes the expansion and accumulation of the MDSC in tumor milieu by engaging A2B receptors on myeloid precursor cells [19]. 3) Inducing anomalous differentiation and weakening the function of antigen presenting cells: ADO bonding with A2B receptor can alter dendritic cells phenotype, decrease the level of tumor antigen presentation, and increase vascular endothelial growth factor (VEGF) production [20, 21]. Taken together, CD73-adenosinergic pathway involves in creating a tumorigenic microenvironment by regulating the tumor itself and immune system (Figure 1).

Numerous *in vitro* studies demonstrated that CD73 expression was associated with tumor proliferation, invasiveness, angiogenesis, metastasis and therapy resistance. But the prognosis role of CD73 in different human solid tumors is controversially according to the following studies. For epithelial ovarian carcinomas, Oh et al. found CD73-high expression predicted better prognosis, lower stage, and higher degree of differentiation [22]. The similar result has also been reported in nonmuscle-invasive urothelial bladder cancer [23]. Nevertheless, other researches in colorectal, gastric, gallbladder, prostate and triple negative breast cancer, proved CD73 was an unfavorable prognostic marker [2, 5, 24, 25]. For purpose of more precisely evaluate the prognostic value of CD73-adenosinergic pathway in solid tumor, a systematic review and meta-analysis was conducted according to previous published studies.

## RESULTS

### Search results and characteristics of eligible studies

**Figure 2:**
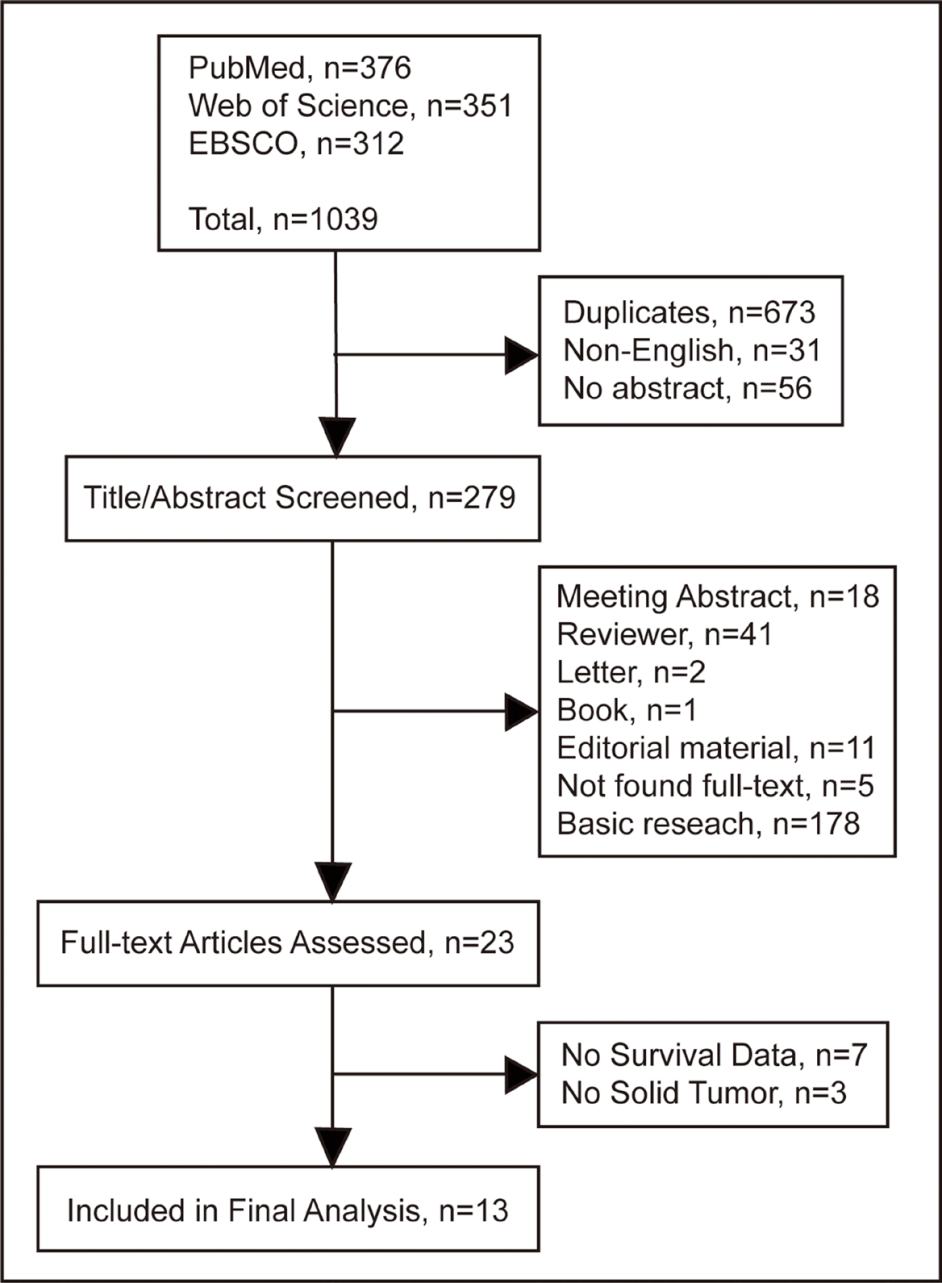
Flow diagram of study identification

Initially, 1039 potential studies were yielded utilizing the electronic databases search, of which 13 articles met the inclusion criteria (Figure 2). Those relevant relevant articles were screened for eligibility by duplication and language, and 760 records were excluded. 256 articles were excluded through title and abstract screening. Another ten publications were further excluded after full-text screening. Finally, 13 publications full met the predefined criteria for this meta-analysis [2–6, 22–29]. Relevant characteristics of the eligible studies are summarized in Table 1.

**Table 1:** Characteristics of included studies

Study, (Author/year)		Country	Tumor type	Tumor stage	Number of Patients	Male/Female	Age, Median (Range)	Follow-up, months (Range)	Survival Data	NOS Score
Wettstein/2015 [23]		Switzerland	Urothelial Bladder Cancer	Nonmuscle Invasive	174	131/43	69.5 (32–92)	110.6 (32.4–226.8)	DFS	8
Turcotte/2015 [5]	A	Canada	High-grade serous ovarian cancer	I–IV	208	0/208	61 (34–89)	36 (1–156)	DFS	7
	B				1581	0/1581	NR	NR	OS	
Leclerc/2015 [4]		Canada	Prostate cancer	I–IV	285	NR	62	108	DFS	7
Yu/2015 [26]		China	Renal cell cancer	I–IV	189	119/70	58 (35–87)	78 (1–118)	OS, DFS	8
Zhang/2015 [24]		China	Rectal cancer	I–IV	90	60/30	64.4 ± 12.5	88.5 (83–98)	OS	7
Xiong/2014 [6]		China	Gallbladder cancer	I–III	67	19/48	54.5 ± 10.6	18	OS	7
Loi/2013 [2]		Australia	Breast cancer	I–IV	6209	0/6209	NR	NR	OS	6
Lu/2013 [25]		China	Gastric cancer	I–IV	68	43/25	49.8 (24–59)	1–81	OS	7
Oh/2012 [22]		Korea	Epithelial ovarian cancer	I–IV	167	0/167	50.3 ± 13.5	1–120	OS, DFS	8
Zhi/2012 [28]		China	Breast cancer	I–IV	2898	0/2898	NR	NR	OS	6
Supernat/2012 [29]		Poland	Breast cancer	I–III	136	0/136	58.4 (27–86)	21.6 (1.2–42)	OS, DFS	7
Wu/2012 [3]	Validation cohort	China	Colorectal cancer	I–IV	135	75/60	59.5 ± 14.0	74.8 ± 36.2	OS	7
	Training cohort	China	Colorectal cancer	I–IV	223	120/103	57.9 ± 13.8	52.0 ± 21.1	OS	7
Cushman/2015 [27]		USA	Colorectal cancer	IV	103	57/46	61.1 (22–83.3)	69.2	OS, DFS	6

The including articles were observational retrospective studies consisting of approximately 12,533 solid tumors patients, which evaluated the association between expression levels of CD73 and survival parameters. The number of participants ranged from 67 to 6209, and the median follow-up ranged from 18 to 110.6 months. 10/13 of the included studies got a quality score ≥ 6 NOS assessment.

### Evaluation of CD73 expression

The description of detection and definition method of CD73 used in eligible researches was generalized in Supplementary Table 1. In those including studies, three studies used *CD73* gene expression, immunohistochemical staining (IHC) was applied in nine studies, and one study (Turcotte et al.) both using gene and protein detection. Among the studies defined CD73 positive, median expression of CD73 staining was 50.77%, and CD73 expression in solid tumors ranged from 26.4% to 74%. Most studies used tumor cell staining for determination of CD73 expression, however, Leclerc et al. and Zhang et al. also used tumor stroma staining.

### CD73 predicts poor OS

**Figure 3:**
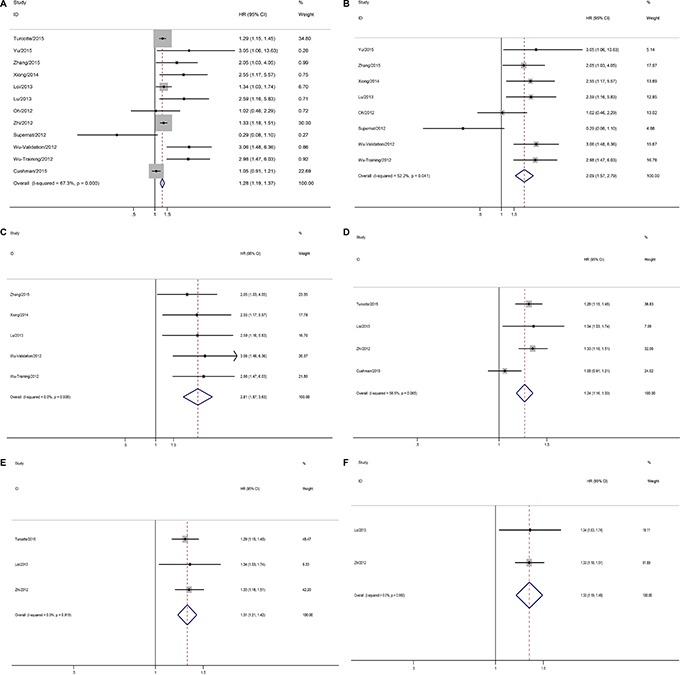
Forest plots showing results of studies on the impact of CD73 expression of overall survival (OS) (**A**) Impact of CD73 expression on OS of patients with solid tumors; (**B**) Impact of CD73 expression by immunohistochemistry (IHC) on OS of patients with solid tumors; (**C**) Impact of CD73 expression by immunohistochemistry (IHC) on OS of patients with gastrointestinal cancer; (**D**) Impact of CD73 expression by *CD73* gene detection on OS of patients with solid tumors; (**E**) Sensitivity analysis was conducted, and excluding Cushman et al., study, the impact of CD73 expression by *CD73* gene detection on OS of patients with solid tumors; (**F**) Impact of CD73 expression by *CD73* gene detection on OS of patients with breast cancer.

Twelve studies provided the association between CD73 and overall survival, including two studies for ovarian cancer, one study for renal cancer, five studies for gastrointestinal cancer, three studies for breast cancer, and one study about prostate cancer. The combined analysis from the published data showed that CD73-high expression was significantly associated with worse OS (pooled HR = 1.28, 95% CI = 1.19–1.37). We also found heterogeneity existed (*I^2^* = 67.3%, *p* = 0.000) (Figure 3A). Next we conducted subgroup meta-analysis according to CD73 detection methods (IHC and gene expression). IHC evaluated in eight studies demonstrated that over-expression of CD73 was correlated with poor OS (pooled HR = 2.09, 95% CI = 1.57–2.79) (Figure 3B). And CD73 gene expression was used in four studies, and high level of *CD73* was also related with worse OS (pooled HR = 1.24, 95% CI = 1.16–1.33). Heterogeneity was also found in this analysis (*I^2^* = 58.5%, *p* = 0.065) (Figure 3D). Sensitivity analysis was conducted, and we found this heterogeneity was influenced by study sample size. After excluding Cushman et al.’s study, the heterogeneity decreased (*I^2^* = 0.0%, *p* = 0.919) (Figure 3E).

Subset analyses based on cancer types were also conducted. Five studies for gastrointestinal cancer using IHC detection demonstrated that CD73 over-expression implied an unfavorable OS (pooled HR = 2.61, 95% CI = 1.87–3.63) (Figure 3C). As well as in breast cancer, CD73-high expression was associated with reduced OS (pooled HR = 1.33, 95% CI = 1.19–1.49) (Figure 3F).

### CD73 implies unfavorable DFS

**Figure 4:**
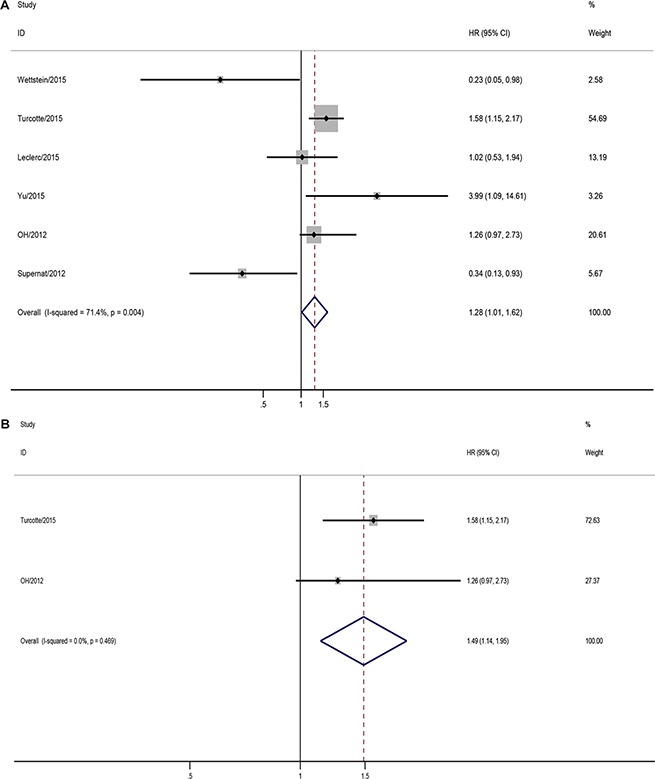
Meta-analysis of impact of CD73 expression on disease free survival (DFS) of patients with solid tumors (**A**) Impact of CD73 expression on DFS of patients with solid tumors; (**B**) Impact of CD73 expression on DFS of patients with ovarian cacner

Six studies assessing CD73 expression by IHC talked about the solid tumors DFS. In total, 1159 patients were in the pooled analysis. CD73 positive expression had borderline association with unfavorable DFS (pooled HR = 1.28, 95% CI = 1.01–1.62), and significant heterogeneity (*I^2^*=71.4%, *p* = 0.004) (Figure 4A). Sensitivity analysis found the heterogeneity was generated from solid tumor categories. And subgroup analysis found CD73 overexpressed in ovarian cancer was significantly associated with poor DFS (pooled HR = 1.49, 95% CI = 1.14–1.95) (Figure 4B).

### Sensitivity analysis and publication bias

**Figure 5:**
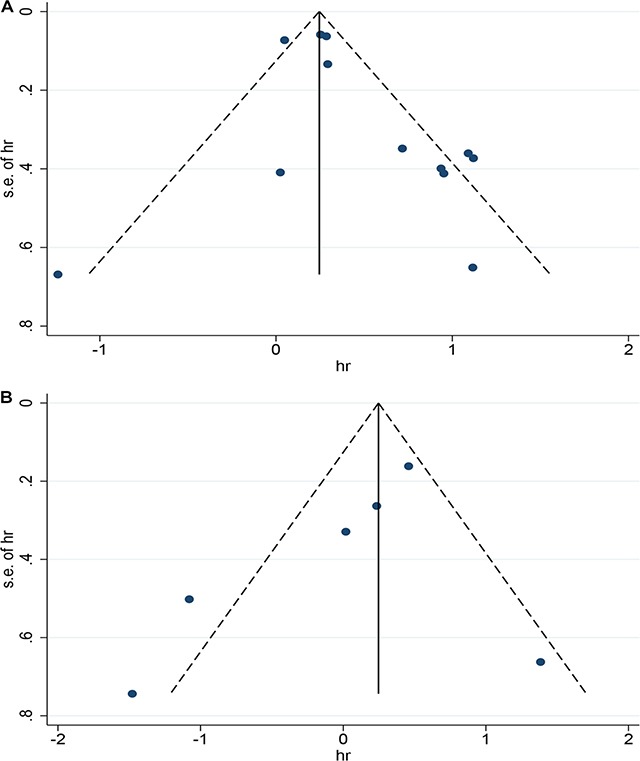
Funnel plot for the evaluation of potential publication bias in the impact of CD73 on overall survival (**A**) and disease free survival (**B**) of patients with solid tumors.

Sensitivity analyses were carried out through sequential removal of individual studies to assess the potential heterogeneity of each study on the pooled HRs (data not shown). Egger’s tests and symmetric funnel plots indicated no evidence of significant publication bias in this meta-analysis (Figure 5). However, because of the limited studies included, there might still have possible publication bias in the current meta-analysis difficult to confirm.

## DISCUSSION

CD73-adenosinergic pathway has been involved in the pathophysiology of substantial solid tumors. CD73 is thought as a key point in this pathway, but the prognostic role across different solid tumors is still in contradiction. This comprehensive meta-analysis of published data was conducted to assess expression of CD73 and their connection with solid tumors prognosis (for studies that evaluated CD73 by IHC or gene detection). We found that CD73-high expression is a marker of poor OS and DFS in solid tumors.

Among the evaluated tumor types, over-expression of CD73 in gastrointestinal cancer was the one mostly linked with a worse outcome. But in breast cancer, the association of CD73 with long-term survival was controversial, and our analysis results found CD73 was boundary linked to worse survival. It may influenced by strong heterogeneity of breast cancer. Supernat et al. using IHC test showed the positive CD73 staining predicts longer DFS and OS on breast cancer. However, this study did not distinguish the breast cancer molecular subtypes [29]. Interestingly, Loi’s group through analyzing 44 publicly available microarray datasets about 6,209 breast cancer samples, they implied *CD73* gene expression was associated with worse prognosis in triple negative breast cancer patients (HR = 1.5, 95% CI = 1–2.1, *p* = 0.029), but not in Luminal type (HR = 0.96, 95% CI = 0.77–1.2, *p* = 0.7) or HER2 positive type(HR = 1.0, 95% CI = 0.71–1.5, *p* = 0.86) [2]. The above two studies indicted CD73 may play different role in different subtypes of breast cancer. All of these suggested that CD73 as a prognostic biomarker for clinical use may depends on the tumor types and subtypes.

As we all known that CD73 expresses in both tumor cells and stromal cells, but the CD73 expression in tumor stromal cells was less well investigated. Only two studies involved the stromal CD73, Leclerc’s study showed that positive CD73 staining in prostate cancer stroma was associated with longer biochemical recurrence free survival in univariate analysis (but not significant in multivariate analysis) [4]. A rectal adenocarcinoma research by Zhang et al. had also indicted that higher stromal CD73 expression was linked to early tumor stages and favorable OS. However our meta-analysis may provide strong supporting evidence for CD73 associated with poor OS and DFS [24]. This contradiction suggested that the prognostic role of CD73 may also influenced by the location of CD73 expression.

CD73-adenosinergic pathway promote tumor progress not only by regulating the tumor cells proliferation and angiogenesis, but also promoting the tumor to form a suppressive milieu by inhibiting CD8+ T cells, NK cells function, and increasing the generation of MDSCs. This involved tumorigenic microenvironment provide basic research evidences for our meta-analysis results that CD73 over-expression was associated with worse survival outcomes in several solid tumors. And reminds us that blocking this protein in CD73-adenosinergic pathway may be a useful therapeutic target for clinical intervention for different solid tumors in future [30]. Recently, many preclinical and phase I trials of anti-CD73 have been performed [11, 31–34] (Table 2). MEDI9447 (a monoclonal antibody specific for the ectoenzyme, CD73) can reduce immunosuppression via increasing CD8+ T cells, inhibiting myeloid-derived suppressor cells and regulatory T cells in the tumor microenvironment, finally inhibit tumor progress. What’ more, Studies also showed that anti-CD73 not only exhibited the activity as a single agent, but also enhanced the activity of PD-1 blockade [31]. So CD73 as therapeutic target is becoming promising and complex.

**Table 2:** Pre-clinical and clinical trials evaluating anti–CD73 therapeutic strategies

Study	Phase/Condition	Experimental arm (s)	Efficiency	Reference
*MedImmune/2016*	*Phase 1(NCT02503774) Advanced Solid Tumors*	*Arm A: MEDI9447* *Arm B: MEDI9447+MEDI4736*	*Recruiting*	www.clinicaltrials.gov
Hay/2015	Mice model CT26 colon cancer	Arm A: Untreated Arm B: Isotype Mix Arm C: MEDI9447 Arm D: Anti-PD1 Arm E: MEDI9447+Anti-PD1	Enhances anti-tumor activity of anti-PD1	[31]
Stagg/2010	Mice model 4T1.2 breast cancer	Arm A: Ig Arm B: anti-CD73 mAb (TY/23)	Inhibits breast tumor growth and metastasis	[30]
Terp/2013	Mice model LM3 hepatocellular cancer	Arm A: anti-CD73 AD2 mAb Arm B: control mAb	Inhibits the ability of circulating tumor cells to extravasate and colonize, leading to inhibition of metastasis	[32]
Allard/2013	Mice model MC38-OVA colon cancer, RM-1 prostate cancer, 4T1.2 breast cancer	Arm A: Ig Arm B: anti-CD73 mAb (TY/23) Arm C: anti-PD-1 mAb (RMP1-14) Arm D: anti-CTLA-4 mAb (UC10-4F10) Arm E: TY/23+RMP1-14 Arm F: TY/23+ UC10-4F10	Enhances the therapeutic activity of anti-PD-1 and anti-CTLA-4 mAbs	[33]
Wang/2011	Mice model ID8 ovarian cancer	Arm A: Untreated Arm B: APCP Arm C: T cells Arm D: T cells+APCP* Arm E: anti-CD73 mAb (TY/23) Arm F: T cells+TY/23	Inhibits tumor growth and augments the efficacy of adoptive T cell therapy	[11]

* APCP: selective inhibitor α,β-methylene adenosine 5′-diphosphate.

Several limitations may exist in our meta-analysis. Firstly, the method and cut-off values for assessing CD73 expression are inconsistent. Secondly, due to the limited number of studies, we were incapable to perform detail subgroup analyses to avoid the tumor heterogeneity, so it may exists some heterogeneity. Finally, this is a literature based analysis, small and negative results researches may not be published, which may account for publication bias.

Despite the above limitations, this study was the first meta-analysis demonstrated that CD73-high expression in solid tumor tissues was associated with a worse prognosis, suggesting that CD73 could be used as a predict biomarker and targeting CD73 might be a promising therapeutic approach for solid tumors. Further data are required for the potential impact of CD73-adenosinergic pathway in tumors, especially under different molecule subtypes and different expression location. If anti-CD73 therapy can be proved effective finally, it might challenge the paradigm of adjuvant therapy for solid tumor. Future research, especially randomized controlled trials (RCT), is desirable to confirm this therapy to reduce cancer mortality.

## MATERIALS AND METHODS

### Literature search and selection criteria

This study based on Preferred Reporting Items for Systematic Reviews and Meta-Analyses guidelines. And according to the existing literature, so ethical committee or institutional review board approvals were not necessary.

Literature search was conducted independently by two investigators via PubMed, Web of Science and EBSCO published between January 1990 and January 2016. The following MeSH terms were used in search strategy: (“NT5E” OR “Ecto-5′-nucleotidase” OR “CD73”) AND (“CANCER” OR “TUMOR” OR “CARCINOMA”). Studies were selected for further analyze based on careful reading of the online titles and abstracts.

Publications meet all of the following criteria were included: a) studies investigated the association between CD73 and prognostic parameters of solid tumor; b) contained sufficient published data to calculate hazard ratio (HR) and 95% confidence interval (95% CI); and c) original research with English full text. While, duplicated articles or overlapped data were excluded.

### Data extraction and assessment of study quality

Two investigators independently evaluated and selected the articles and extracted information in a standardized manner and subsequently resolved disagreements by discussion with another author. The following relevant data were extracted from eligible articles: first author’s name, year, country; number of analyzed patients, assessment methods for CD73 expression, follow-up time, tumor site and stage, and most importantly, the long-term survival (e.g., overall survival (OS) and disease-free survival (DFS)). However, the cut-off value for CD73 varied among including publications, so CD73-high expression was defined refer to the original articles. In order to avoid bias, the HR was extracted preferentially from multivariable analyses. If not available, HR from univariate analyses also extracted. Kaplan–Meier curves were evaluated using GetData Graph Digitizer 2.26 (http://getdatagraph-digitizer.com) for the articles did not provide survival data directly.

Two independent authors conducted the quality assessment for each eligible study using Newcastle–Ottawa Scale (NOS). NOS score higher than six were indicated as high-quality studies.

### Statistical analysis

Survival outcomes were the primary end-points in this study, so the data synthesized using HR and 95% CI to evaluate the impact of CD73 expression on long-term survival of solid tumor. The heterogeneity of pooled data was measured using the Cochran *Q*-test and I-squared test. When *I^2^* < 50% and *p* > 0.10 were considered as no heterogeneity and a fixed-effects model (Mantel-Haenszel) was performed. Otherwise, random-effects model (DerSimonian and Laird) was adopted. The sources of inter-study heterogeneity were explored using subgroup analysis and sensitivity analysis. A funnel plot with Begg’s and Egger’s test was applied to assess the potential publication bias.

Statistical analyses were performed using STATA 12.0 software (STATA Corporation, College Station, TX, http://www.stata.com). And statistical significance was defined as *p*-value < 0.05.

## SUPPLEMENTARY MATERIALS FIGURES AND TABLES





## References

[1] Antonioli L, Blandizzi C, Pacher P, Haskó G (2013). Immunity, inflammation and cancer: a leading role for adenosine. Nat Rev Cancer.

[2] Loi S, Pommey S, Haibe-Kains B, Beavis PA, Darcy PK, Smyth MJ, Stagg J (2013). CD73 promotes anthracycline resistance and poor prognosis in triple negative breast cancer. P Natl Acad Sci USA.

[3] Wu XR, He XS, Chen YF, Yuan RX, Zeng Y, Lian L, Zou YF, Lan N, Wu XJ, Lan P (2012). High expression of CD73 as a poor prognostic biomarker in human colorectal cancer. J Surg Oncol.

[4] Leclerc BG, Charlebois R, Chouinard G, Allard B, Pommey S, Saad F, Stagg J (2015). CD73 Expression Is an Independent Prognostic Factor in Prostate Cancer. Clin Cancer Res.

[5] Turcotte M, Spring K, Pommey S, Chouinard G, Cousineau I, George J, Chen GM, Gendoo DM, Haibe-Kains B, Karn T, Rahimi K, Le Page C, Provencher D (2015). CD73 is associated with poor prognosis in high-grade serous ovarian cancer. Cancer Res.

[6] Xiong L, Wen Y, Miao X, Yang Z (2013). NT5E and FcGBP as key regulators of TGF-1-induced epithelial–mesenchymal transition (EMT) are associated with tumor progression and survival of patients with gallbladder cancer. Cell Tissue Res.

[7] Gao ZW, Dong K, Zhang HZ (2014). The roles of CD73 in cancer. Biomed Res Int.

[8] Spychala J, Lazarowski E, Ostapkowicz A, Ayscue LH, Jin A, Mitchell BS (2004). Role of Estrogen Receptor in the Regulation of Ecto-5′-Nucleotidase and Adenosine in Breast Cancer. Clinical Clin Cancer Res.

[9] Antonioli L, Hasko G, Fornai M, Colucci R, Blandizzi C (2014). Adenosine pathway and cancer: where do we go from here?. Expert Opin Ther TA.

[10] Antonioli L, Yegutkin GG, Pacher P, Blandizzi C, Haskó G (2016). Anti-CD73 in Cancer Immunotherapy: Awakening New Opportunities. Trends in Cancer.

[11] Wang L, Fan J, Thompson LF, Zhang Y, Shin T, Curiel TJ, Zhang B (2011). CD73 has distinct roles in nonhematopoietic and hematopoietic cells to promote tumor growth in mice. J Clin Invest.

[12] Gao ZW, Wang HP, Lin F, Wang X, Long M, Zhang HZ, Dong K (2017). CD73 promotes proliferation and migration of human cervical cancer cells independent of its enzyme activity. BMC cancer.

[13] Antonioli L, Pacher P, Vizi ES, Hasko G (2013). CD39 and CD73 in immunity and inflammation. Trends Mol Med.

[14] Cekic C, Day YJ, Sag D, Linden J (2014). Myeloid expression of adenosine A2A receptor suppresses T and NK cell responses in the solid tumor microenvironment. Cancer Res.

[15] Gaudreau PO, Allard B, Turcotte M, Stagg J (2016). CD73-adenosine reduces immune responses and survival in ovarian cancer patients. Oncoimmunology.

[16] Németh ZH, Lutz CS, Csóka B, Deitch EA, Leibovich SJ, Gause WC, Tone M, Pacher P, Vizi ES, Haskó G (2005). Adenosine Augments IL-10 Production by Macrophages through an A2B Receptor-Mediated Posttranscriptional Mechanism. J Immunol.

[17] Csóka B, Selmeczy Z, Koscsó B, Németh ZH, Pacher P, Murray PJ, Kepka-Lenhart D, Morris SM, Gause WC, Leibovich SJ, Haskó G (2012). Adenosine promotes alternative macrophage activation via A2A and A2B receptors. FASEB J.

[18] György Haskó PP (2012). Regulation of Macrophage Function by Adenosine. Arterioscler Thromb Vasc Biol.

[19] Ryzhov S, Novitskiy SV, Goldstein AE, Biktasova A, Blackburn MR, Biaggioni I, Dikov MM, Feoktistov I (2011). Adenosinergic Regulation of the Expansion and Immunosuppressive Activity of CD11b+Gr1+ Cells. J Immunol.

[20] Young A, Ngiow SF, Barkauskas DS, Sult E, Hay C, Blake SJ, Huang Q, Liu J, Takeda K, Teng MW, Sachsenmeier K, Smyth MJ (2016). Co-inhibitionof CD73 and A2AR Adenosine Signaling Improves Anti-tumor Immune Responses. Cancer Cell.

[21] Novitskiy SV, Ryzhov S, Zaynagetdinov R, Goldstein AE, Huang Y, Tikhomirov OY, Blackburn MR, Biaggioni I, Carbone DP, Feoktistov I, Dikov MM (2008). Adenosine receptors in regulation of dendritic cell differentiation and function. Blood.

[22] Oh HK, Sin JI, Choi J, Park SH, Lee TS, Choi YS (2012). Overexpression of CD73 in epithelial ovarian carcinoma is associated with better prognosis, lower stage, better differentiation and lower regulatory T cell infiltration. J Gynecol Oncol.

[23] Wettstein MS, Buser L, Hermanns T, Roudnicky F, Eberli D, Baumeister P, Sulser T, Wild P, Poyet C (2015). CD73 Predicts Favorable Prognosis in Patients with Nonmuscle-Invasive Urothelial Bladder Cancer. Dis Markers.

[24] Zhang B, Song B, Wang X, Chang XS, Pang T, Zhang X, Yin K, Fang GE (2015). The expression and clinical significance of CD73 molecule in human rectal adenocarcinoma. Tumour Biol.

[25] Lu XX, Chen YT, Feng B, Mao XB, Yu B, Chu XY (2013). Expression and clinical significance of CD73 and hypoxia-inducible factor-1alpha in gastric carcinoma. World J Gastroenterol.

[26] Yu Y, Wang W, Song L, Hu W, Dong C, Pei H, Zhou G, Yue Z (2015). Ecto-5′-nucleotidase expression is associated with the progression of renal cell carcinoma. Oncology Letters.

[27] Cushman SM, Jiang C, Hatch AJ, Shterev I, Sibley AB, Niedzwiecki D, Venook AP, Owzar K, Hurwitz HI, Nixon AB (2014). Gene Expression Markers of Efficacy and Resistance to Cetuximab Treatment in Metastatic Colorectal Cancer: Results from CALGB 80203 (Alliance). Clin Cancer Res.

[28] Zhi X, Wang Y, Yu J, Yu J, Zhang L, Yin L, Zhou P (2012). Potential prognostic biomarker CD73 regulates epidermal growth factor receptor expression in human breast cancer. IUBMB Life.

[29] Supernat A, Markiewicz A, Wełnicka-Jaśkiewicz M, Seroczyńska B, Skokowski J, Sejda A, Szade J, Czapiewski P, Biernat W, Żaczek A (2012). CD73 Expression as a Potential Marker of Good Prognosis in Breast Carcinoma. Appl Immunohisto M M.

[30] Stagg J (2012). The double-edge sword effect of anti-CD73 cancer therapy. Oncoimmunology.

[31] Carl Hay KM, McGlinchey K, Fuhrmann S, Huang Q, Sult E, Rothstein R, Rios-Doria J, Durham N, Poon E, Hammond S, Stewart R, Herbst R, Hollingsworth R, Sachsenmeier K (2015). MEDI9447: enhancing anti-tumor immunity by targeting CD73 In the tumor microenvironment. Cancer Res.

[32] Terp MG, Olesen KA, Arnspang EC, Lund RR, Lagerholm BC, Ditzel HJ, Leth-Larsen R (2013). Anti-human CD73 monoclonal antibody inhibits metastasis formation in human breast cancer by inducing clustering and internalization of CD73 expressed on the surface of cancer cells. J Immunol.

[33] Allard B, Pommey S, Smyth MJ, Stagg J (2013). Targeting CD73 enhances the antitumor activity of anti-PD-1 and anti-CTLA-4 mAbs. Clin Cancer Res.

[34] Stagg J, Divisekera U, McLaughlin N, Sharkey J, Pommey S, Denoyer D, Dwyer KM, Smyth MJ (2010). Anti-CD73 antibody therapy inhibits breast tumor growth and metastasis. P Natl Acad Sci USA.

