# Supplementary material for: Prognositic value of CD73-adenosinergic pathway in solid tumor: A meta-analysis and systematic review
Source: Oncotarget. 2017 Apr 6;8(34):57327–36. doi: 10.18632/oncotarget.16905 (PMC5593644; doi:10.18632/oncotarget.16905)
Supplement: Supplementary file 1 [file oncotarget-08-57327-s001.pdf]

Prognositic value of CD73-adenosinergic pathway in solid tumor: A meta-analysis and systematic review

Supplementary Materials

Supplementary Table 1: Evaluation of human CD73 expression in the selected studies.
See Supplementary_Table_1
