# Supplementary material for: Prognositic value of CD73-adenosinergic pathway in solid tumor: A meta-analysis and systematic review
Source: Oncotarget. 2017 Apr 6;8(34):57327–36. doi: 10.18632/oncotarget.16905 (PMC5593644; doi:10.18632/oncotarget.16905)
Supplement: Supplementary file 2 [file oncotarget-08-57327-s002.doc]

**Supplementary Table 1: Evaluation of human CD73 expression in the selected studies**

| **Study, (Author/year)** | | **Tumor type** | **Test methods** | **CD73 location** | **CD73 (+)%** | **Antibody used for the**  **evaluation of CD73 by IHC** | **Cut-off for overexpression** |
| --- | --- | --- | --- | --- | --- | --- | --- |
| Wettstein/2015 [23] | | Urothelial Bladder Cancer | IHC | Tumor cell | 26.4 | Anti-NT5E rabbit polyclonal antibody, Sigma, USS | Using a semiquantitative three-scale scoring system ranging from 0 to 2+ (score 0: no staining; score 1+: weak staining; score 2+: strong staining). CD73 low-expression group (containing scores 0 and 1+) and a CD73 high-expression group (containing score 2+). |
| Turcotte/2015  [5] | A | High-grade serous ovarian cancer | IHC | Tumor cell | NR | Clone 1D7, Abcam, UK | Higher than 20% CD73 expression was used as an arbitrary cutoff. |
|  | B |  | Gene microarray [Australian Ovarian Cancer Study (AOCS; GSE9899) cohort] | Tumor cell | NR | None | Expression level higher than a threshold (median + 0.5*median absolute deviation) were classified as “High” and samples lower than a threshold (median – 0.5*mad) were classified as “Low”. |
| Leclerc/2015 [4] | | Prostate cancer | IHC and IF | Tumor cell and Tumor stroma | NR | Clone 1D7, Abcam, UK | CD73 expression higher than median values was used as an arbitrary cutoff. |
| Yu/2015 [26] | | Renal cell cancer | IHC | Tumor cell | 39.68 | cat. no. ab115289; Abcam, UK | A semi-quantitative scoring system based on the staining intensity, with low, weak, moderate and strong staining being classified as levels 0, 1, 2 and 3, respectively, and the percentage of cells with intense staining, with levels 0, 1, 2 and 3 being defined as no cells positively stained, <25% cells stained, 26-50% cells stainedand>50% cells stained, respectively. For each slide, three fields were evaluated. CD73 low-expression group (containing scores 0) and a CD73 high-expression group (containing score 1, 2, 3). |
| Zhang/2015 [24] | | Rectal cancer | IHC and IF | Tumor cell and Tumor stroma | [Tumor cell]: 52.22; [Tumor stroma]: 53.33 | rabbit. ab133582; Abcam, UK | CD73 expression higher than median values was used as an arbitrary cutoff. |
| Xiong/2014 [6] | | Gallbladder cancer | IHC | Tumor cell | 54.6 | EnVision™Detection Kit | The strength of staining was rated on a scale of 1 to 3. A score of 1 represents little to no positive staining or uncertain weak staining; 2 represents weak to moderate staining; 3 represents moderate to strong staining. A section was determined as positive for NT5E when the percent of positively stained cells was >10 % and staining strength >2. The few sections where percent positive staining was 5 % to 10 % and staining strength was 3 were also regarded as positive. |
| Loi/2013 [2] | | Breast cancer | Gene microarray [GEO, ArrayExpress, Stanford Microarray Database, MD Anderson Cancer Center Microarray database, University of North Carolina database, and Rosetta Inpharmatics] | Tumor cell | NR | None | NR |
| Lu/2013 [25] | | Gastric cancer | IHC | Tumor cell | 45.59 | ab71322 Abcam, UK | The percentage of positive cells was scored 0 for staining of < 1%, 1 for staining of 2%-25%, 2 for staining of 26%-50%, 3 for staining of 51%-75%, and 4 for staining > 75% of the cells examined. Staining intensity was calculated, no coloring, slightly yellow, brown yellow and tan stains were marked as 0, 1, 2 and 3. Finally, we calculated the product of staining intensity and positive cell percentage: ≤5 was defined as negative and ≥6 as positive. |
| Oh/2012 [22] | | Epithelial ovarian cancer | IHC | Tumor cell | 70.1 | ab91086; Abcam, UK | The staining was scored as 0 if no cancer cells were reactive, 1 if  staining was weakly positive in <1/3 of cancer cells, 2 if staining was weakly positive in >2/3 of cancer cells, or strongly positive in >1/3 of cancer cells, and 3 if staining was weakly positive in most of cancer cells, or strongly positive in >2/3 of cancer cells |
| Zhi/2012 [28] | | Breast cancer | Gene microarray [www.kmplot.com] | Tumor cell | 49.86 | None | NR |
| Supernat/2012 [29] | | Breast cancer | IHC | Tumor cell | 74 | clone IE9; Santa Cruz Biotechnology | Immunostaining was scored following 4-step scale (0 = negative, 1 =weak, 2 =intermediate, 3 =strong). The percentage of stained cells in a quantitative manner (0% to 100%) was also determined. Positive CD73 status was considered as the score higher then median value. |
| Wu/2012 [3] | Validation cohort | Colorectal cancer | IHC | Tumor cell | 50.37 | mouse monoclonal CD73 antibody; Abcam, UK | X-tile program to determine cutoff value for CD73 expression index. According to the X-tile program, the training cohort was divided into low expression and high expression populations based on a cut-point of 5.9 |
|  | Training cohort |  |  |  | 44.84 |  |  |
| Cushman/2015 [27] | | Colorectal cancer | TaqMan quantitative PCR | Tumor cell | NR | None | NR |
